# Hypercapnic acidosis attenuates pulmonary epithelial stretch-induced injury via inhibition of the canonical NF-κB pathway

**DOI:** 10.1186/s40635-016-0081-6

**Published:** 2016-03-22

**Authors:** Shahd Horie, Bilal Ansari, Claire Masterson, James Devaney, Michael Scully, Daniel O’Toole, John G. Laffey

**Affiliations:** Discipline of Anaesthesia, School of Medicine, Clinical Sciences Institute, National University of Ireland, Galway, Ireland; Regenerative Medicine Institute, National University of Ireland, Galway, Ireland; Department of Anesthesia, Critical Illness and Injury Research Centre, Keenan Research Centre for Biomedical Science, St Michael’s Hospital, University of Toronto, Toronto, Canada

**Keywords:** Acute respiratory distress syndrome, Inflammation, Ventilation-induced lung injury, Repair, Hypercapnic acidosis, Nuclear factor kappa-B

## Abstract

**Background:**

Hypercapnia, with its associated acidosis (HCA), is a consequence of respiratory failure and is also seen in critically ill patients managed with conventional “protective” ventilation strategies. Nuclear factor kappa-B (NF-κB), a pivotal transcription factor, is activated in the setting of injury and repair and is central to innate immunity. We have previously established that HCA protects against ventilation-induced lung injury in vivo, potentially via a mechanism involving inhibition of NF-κB signaling. We wished to further elucidate the role and mechanism of HCA-mediated inhibition of the NF-κB pathway in attenuating stretch-induced injury in vitro.

**Methods:**

Initial experiments examined the effect of HCA on cyclic stretch-induced inflammation and injury in human bronchial and alveolar epithelial cells. Subsequent experiments examined the role of the canonical NF-κB pathway in mediating stretch-induced injury and the mechanism of action of HCA. The contribution of pH versus CO_2_ in mediating this effect of HCA was also examined.

**Results:**

Pulmonary epithelial high cyclic stretch (22 % equibiaxial strain) activated NF-κB, enhanced interleukin-8 (IL-8) production, caused cell injury, and reduced cell survival. In contrast, physiologic stretch (10 % strain) did not activate inflammation or cause cell injury. HCA reduced cyclic mechanical stretch-induced NF-κB activation, attenuated IL-8 production, reduced injury, and enhanced survival, in bronchial and alveolar epithelial cells, following shorter (24 h) and longer (120 h) cyclic mechanical stretch. Pre-conditioning with HCA was less effective than when HCA was applied after commencement of cell stretch. HCA prevented the stretch-induced breakdown of the NF-κB cytosolic inhibitor IκBα, while IκBα overexpression “occluded” the effect of HCA. These effects were mediated by a pH-dependent mechanism rather than via CO_2_ per se.

**Conclusions:**

HCA attenuates adverse mechanical stretch-induced epithelial injury and death, via a pH-dependent mechanism that inhibits the canonical NF-κB activation by preventing IκBα breakdown.

**Electronic supplementary material:**

The online version of this article (doi:10.1186/s40635-016-0081-6) contains supplementary material, which is available to authorized users.

## Background

Acute respiratory distress syndrome (ARDS) is a devastating disorder that has high mortality rates [1]. ARDS is characterized by pulmonary edema, hypoxemia, and severe inflammation of the lung parenchyma [1]. Mechanical ventilation, while life-saving, can cause additional lung damage due to repetitive cyclic mechanical stretch of the lung resulting in increased inflammation and lung tissue injury—this is termed “ventilator induced lung injury” (VILI) [2]. Protective ventilator strategies, that reduce the potential for excessive lung stretch, have been demonstrated to improve outcome [3] and now constitute the standard of care for these patients [4]. However, reducing tidal and minute ventilation results in less effective CO_2_ clearance. The resulting hypercapnic acidosis (HCA) is termed “permissive hypercapnia” and is not traditionally considered to have an active role [5, 6].

Previous pre-clinical studies, from our group and others, have demonstrated that HCA may exert protective effects in models of ARDS induced by free radicals [7], endotoxin [8], both primary [9, 10] and secondary [11] ischemia-reperfusion, and high lung stretch [12, 13]. HCA can also exert deleterious effects, including delayed plasma membrane re-sealing [14] and reduced pulmonary epithelial wound closure [15]. While HCA exerts beneficial effects in early lung injury [16–18] and systemic sepsis [19, 20], prolonged HCA may retard bacterial killing and worsen pneumonia-induced lung injury [21, 22].

These effects of HCA may be mediated at least in part via inhibition of nuclear factor kappa-B (NF-κB), a pivotal regulator of genes central to lung injury, inflammation, and repair [23–26]. Our group has demonstrated that HCA attenuates VILI severity in vivo, potentially via inhibition of NF-κB signaling [27]. HCA also attenuated cyclic mechanical stretch-induced inflammation injury and reduced NF-κB activation [27]. Most recently, we have demonstrated the effects and mechanisms by which HCA modulates the canonical NF-κB pathway in sepsis-induced lung injury [28].

In the current studies, we wished to further elucidate the role of the NF-κB pathway in mediating the protective effects of HCA in stretch-induced injury. Specifically, we hypothesized that (1) HCA would attenuate stretch-induced cell inflammation and injury and improve cell survival in both the bronchial and alveolar epithelium; (2) that the protective effects of HCA seen in early stretch would persist in the setting of prolonged cyclic mechanical stretch; (3) that the effects of HCA on stretch would be mediated via inhibition of the NF-κB pathway; and (4) the effect of HCA would be mediated by a pH (i.e. acidosis) rather than a CO_2_ effect per se.

## Methods

### Pulmonary epithelial cell cultures

Human bronchial epithelial cells (HBE and BEAS-2B lines) and type II alveolar A549 cells were used in all experiments. BEAS-2B cells (ATCC®, Manassas, VA, USA) are a cell line derived from normal bronchial epithelium, immortalized using a replication-defective SV40/adenovirus 12 hybrid, and cloned. These cells were subcultured in growth medium (Dulbecco’s Modified Eagle’s Medium/Nutrient F12 Ham; Sigma-Aldrich Co., Dublin, Ireland), supplemented with 10 % fetal calf serum, penicillin G (100 U/mL) and streptomycin (100 μg/mL) at 37 °C in a humidified incubator saturated with a gas mixture containing 5 % CO_2_ in air, and used at passages 5–15. The 16HBE14o-(HBE) transformed epithelial cell line was a gift from D. Gruender (University of Vermont, Burlington, VT, USA) as cryopreserved passage 1 culture and used at passages 4–14. These are virally immortalized primary human cells which form correctly polarized cell layers in vitro [29]. These cells were subcultured into growth medium (Minimum Essential Medium, Alpha Modification; Sigma-Aldrich Co.), supplemented with 10 % fetal calf serum, penicillin G (100 U/ml) and streptomycin (100 μg/ml).

A549 cells were purchased from The European Collection of Cell Cultures (Porton Down, UK) as cryopreserved 90-passage culture and used at passages 91–95. All A549 cells and derivatives were passaged in growth medium (RPMI-1640; Sigma-Aldrich Co.), supplemented with 10 % fetal calf serum, penicillin G (100 U/mL), and streptomycin (100 μg/mL) at 37 °C in a humidified incubator saturated with a gas mixture containing 5 % CO_2_ in air. In some experiments, the A549/NF-κB-luc cell line, which incorporates a chromosomally integrated luciferase reporter of NF-κB transcription factor activity, was used (P/N LR0051; Panomics, Fremont, CA, USA). To obtain this cell line, A549 cells were co-transfected with pNF-κB-luc and pHyg followed by hygromycin selection. Hygromycin-resistant cell clones were selected using a functional tumor necrosis factor-α (TNF-α) assay that induces luciferase activity. In another experimental series, the A549/NF-κB-luc cell line was stably transfected with an IκBα super-repressor under the control of a chicken β-actin promoter and then selected with G418 antibiotic (Sigma-Aldrich Co.). This super-repressor ensures that IκBα cannot be phosphorylated and hence canonical NF-κB activation is inhibited. Clones were evaluated based on their ability to prevent IL-1β (10 ng/ml) induction of the luciferase reporter (Additional file 1: Figure S1).

### Pulmonary epithelial cyclical stretch injury model

HBE, BEAS-2B or A549/NF-κB-luc cells were seeded to Bioflex six-well plates (Flexcell International, Hillsborough, NC, USA) at 6 × 10^4^ cells/cm^2^, incubated for 48 h, and re-fed with fresh complete medium. They were then mounted onto the Flexcell FX-4000 T® Tension Plus® baseplate (Flexcell International) where they were pre-conditioned in either normocapnic (5 % CO_2_) or hypercapnic conditions (15 % CO_2_) for 1 h before being subjected to 22 % equibiaxial stretch at a frequency of 0.1 Hz for 24 and 120 h under their respective conditions. Non-stretched cells under identical atmospheric conditions were used as controls for these experiments, based on our demonstration that physiologic stretch did not produce any evidence of cell inflammation or injury (Additional file 1: Figure S2).

### Experimental design

#### Series 1–2: effect of HCA on bronchial epithelial stretch-induced injury

HBE (series 1) and BEAS-2B (series 2) confluent epithelial layers were equilibrated in normocapnia or HCA and then subjected to injurious cyclic stretch (i.e., 22 % equibiaxial stretch at a frequency of 0.1 Hz in the Flexcell FX-4000T®) for 24 h. The potential for HCA to attenuate stretch-induced inflammation, maintain cell membrane integrity, and enhance cell survival in HBE and BEAS-2B epithelial layers was then determined.

#### Series 3–4: effect of HCA on alveolar cell stretch injury

Confluent alveolar epithelial A549/NF-κB-luc cell layers were equilibrated in normocapnia or HCA and then subjected to injurious cyclic stretch for 24 (series 3), or 120 (series 4) h, and the effect of HCA was assessed as described above.

#### Series 5: effect of pre- versus post-conditioning with HCA

Confluent A549/NF-κB-luc cell layers were allocated to normocapnia, HCA pre-incubation followed by normocapnia during cyclic stretch (“HCA-pre”), HCA at the commencement of cyclic stretch (“HCA-post”), or HCA prior to and during stretch (“HCA-combined”). All epithelial layers were subjected to cyclic stretch for 120 h.

#### Series 6: mechanism of action of HCA on stretch-induced NF-κB

The effect of cyclic stretch and HCA on inhibitory-κB-alpha (IκBα), the canonical NF-κB cytosolic inhibitor, was examined. Briefly, A549 monolayers were equilibrated in normocapnia or HCA and then subjected to cyclic stretch for 0, 30, 60, and 120 min, and cytosolic concentrations of IκBα were determined.

The potential for IκBα overexpression to attenuate stretch injury and “occlude” the effects of HCA was then determined. Stably transfected A549/NF-κB-luc cells overexpressing IκBα (methods described above) were seeded to laminin coated Bioflex six-well plates at 6 × 10^4^ cells/cm^2^ and left to reach confluent monolayers for 48 h. These cells were then washed, re-fed, and pre-conditioned as described above before being subjected to injurious cyclic stretch for 120 h.

#### Series 7: acidosis versus CO_2_ on stretch-induced epithelial injury

Metabolic acidosis was produced by adding strong acid (0.02 M Hydrochloric acid) to titrate media pH to that seen with HCA conditions, i.e., 7.1, when incubated in normocapnia. Buffered hypercapnia (BHA) was produced by buffering media pH to normal under hypercapnic conditions using 0.04 M sodium bicarbonate. Finally sodium chloride (either 0.04 or 0.02 M) was added to all groups to ensure that all groups were equi-osmolar. These groups were then subjected to injurious cyclic stretch as described above.

### Assessment of NF-κB activity, inflammation, and cell viability

At the end of each experiment, medium was harvested and the cells scraped from each well into 1 mL of phosphate buffered saline (PBS). Cells were pelleted at 400×*g* for 5 min and resuspended in 1 mL of fresh PBS.

#### NF-κB activity

Five hundred microliters of intact harvested cells were pelleted at 400×*g* for 5 min and resuspended in 100 μL of Reporter Lysis Buffer (Promega Corp., Madison, WI, USA), before being subjected to a freeze/thaw cycle. Forty microliters of cell lysate was mixed with 40 μL of Bright-Glo luciferase substrate (Promega Corp.) and luminescence assessed in a VICTOR™ X plate reader (Perkin Elmer, Waltham, MA, USA) [15].

#### Cytosolic IκBα assessment

Three hundred microliters of intact-harvested cells were pelleted at 400×*g* for 5 min and resuspended in 50 μL of 1× Laemmli SDS-PAGE buffer (0.1 % 2-mercaptoethanol, 0.0005 % bromophenol blue, 10 % glycerol, 2 % SDS, 2 %, 63 mM Tris-HCl pH 6.8) and heated to 100 °C for 2 min. Proteins were separated on polyacrylamide gels (Pierce Biotechnology, Rockford, IL, USA), transferred to nitrocellulose membranes, blocked, and probed using anti-total IκBα (Cell Signaling Technology) and secondary-HRP (Sigma-Aldrich Co.) antibodies in 5 % (*w*/*v*) milk powder-PBS. Membranes were soaked in chemiluminescent substrate (Pierce Biotechnology) and imaged.

For IκBα ELISA, cells were scraped into 1 mL of ice-cold PBS, pelleted at 500×*g* for 5 m and resuspended in 100 μL of 1 % Triton (*v*/*v*) in PBS with protease inhibitors (Pierce protease inhibitor mini tablets, EDTA-free; Fisher Scientific Ireland Ltd, Dublin, Ireland). A BCA protein assay was performed and equal quantities of protein added to a PathScan® Total IκBα Sandwich ELISA (Cell Signaling Technology, Danvers, MA, USA) as per manufacturer’s instructions. Total IκBα present was expressed as fold of the control no-stretch group.

#### Viability assay

Fifty microliters of intact harvested cells were added to a 96-well plate. One hundred microliters of methylthiazolyldiphenyl-tetrazolium bromide (MTT; Sigma-Aldrich Co.) solution (100 μg/mL in complete medium) was added to each well, and the plate returned to a tissue culture incubator (5 % CO_2_) for 2 h. The media and MTT solution was removed, 100 μL of dimethyl sulfoxide (DMSO) was added to each well, the plates were left on an orbital mixer for 30 min, and absorbances read in a VICTOR™ X plate reader at 550 nm wavelength [30].

#### LDH assay

Epithelial injury was assessed by measuring lactate dehydrogenase (LDH), an intracellular enzyme that when present in the medium reflects the extent of epithelial cell damage and lysis. Fifty microliters of harvested medium was used with the CytoTox 96 Non-Radioactive Cytotoxicity Assay Kit (Promega Corp.) to assess LDH release.

#### IL-8 ELISA

Epithelial inflammation was assessed by quantifying the secretion of the NF-κB-dependent cytokine IL-8. The medium was assessed for cellular secretion of IL-8 using an IL-8 sandwich ELISA DuoSet kit (R&D Systems Inc., Minneapolis, MN, USA) as per manufacturer’s instructions.

### Data presentation and analysis

All data was analyzed using a one-way analysis of variance, with group as the factor. Post hoc analysis was carried out with the Student-Newman-Keuls test or Mann-Whitney *U* test with the Bonferroni correction for multiple comparisons, as appropriate. The assumptions underlying all models were checked using suitable residual plots. A *p* value of <0.05 was considered statistically significant.

## Results

### HCA inhibits bronchial epithelial stretch-induced injury

**Fig. 1:**
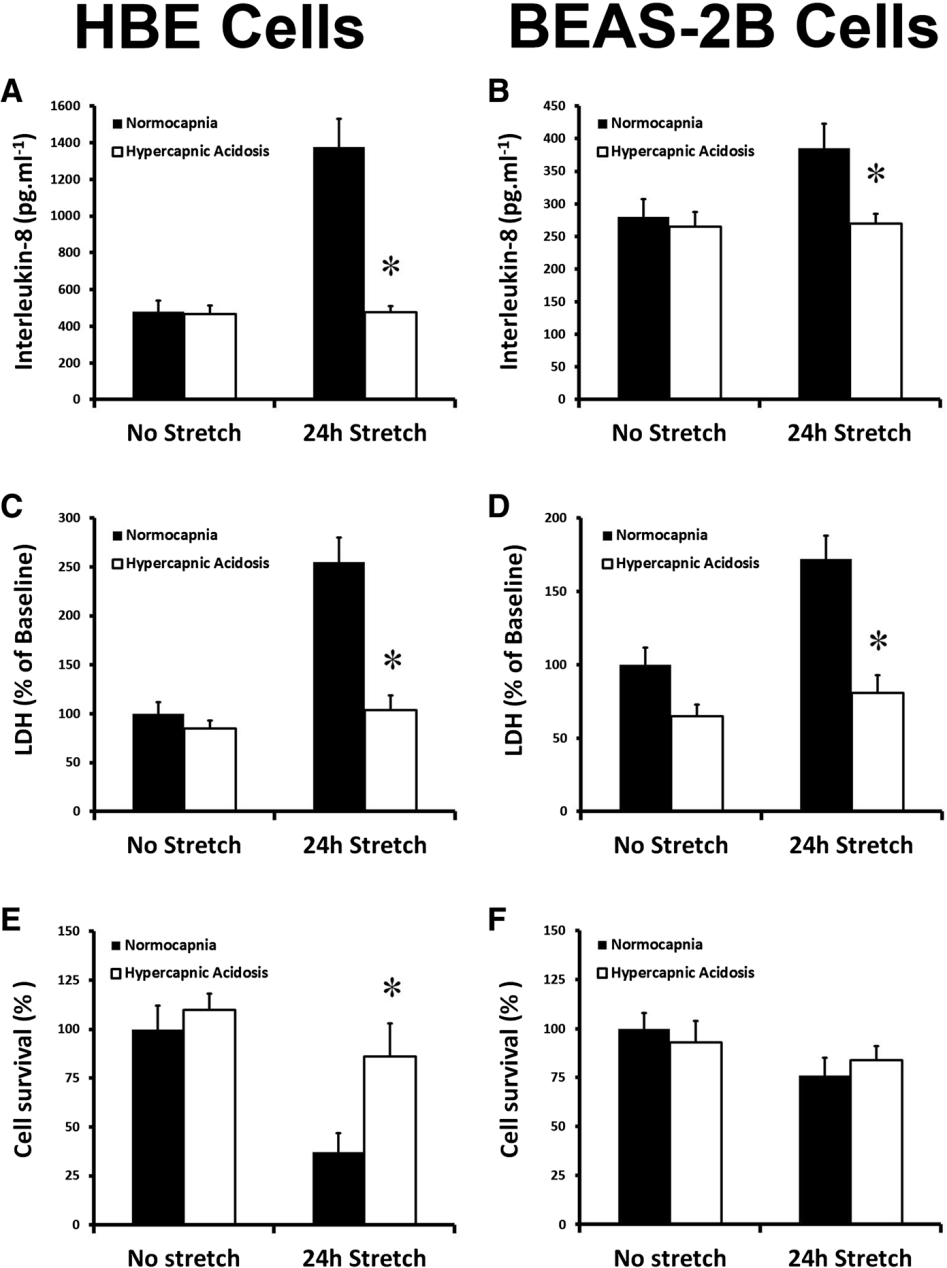
HCA inhibits bronchial epithelial stretch-induced injury. Hypercapnic acidosis decreased 24-h stretch-induced interleukin-8 secretion (**a**, **b**), preserved membrane integrity as assessed by epithelial LDH leakage (**c**, **d**), and maintained cell viability (**e**, **f**) in human bronchial epithelial cells and BEAS-2B cells, respectively. *Note:* *P < 0.05 versus normocapnia for each condition

Twenty-four hours of cyclic cell stretch induced the release of pro-inflammatory IL-8 in both HBE (Fig. 1a) and BEAS-2B (Fig. 1b) bronchial epithelial layers, and this was inhibited by HCA. HCA abolished cell stretch-induced cell membrane injury and rupture, as reflected by medium LDH concentrations, in both HBE (Fig. 1c) and BEAS-2B (Fig. 1d) epithelial bronchial cultures. HCA also attenuated cell stretch-induced cell death, in HBE (Fig. 1e) but not in BEAS-2B (Fig. 1f) epithelial bronchial cultures.

### HCA inhibits prolonged alveolar epithelial stretch-induced injury

**Fig. 2:**
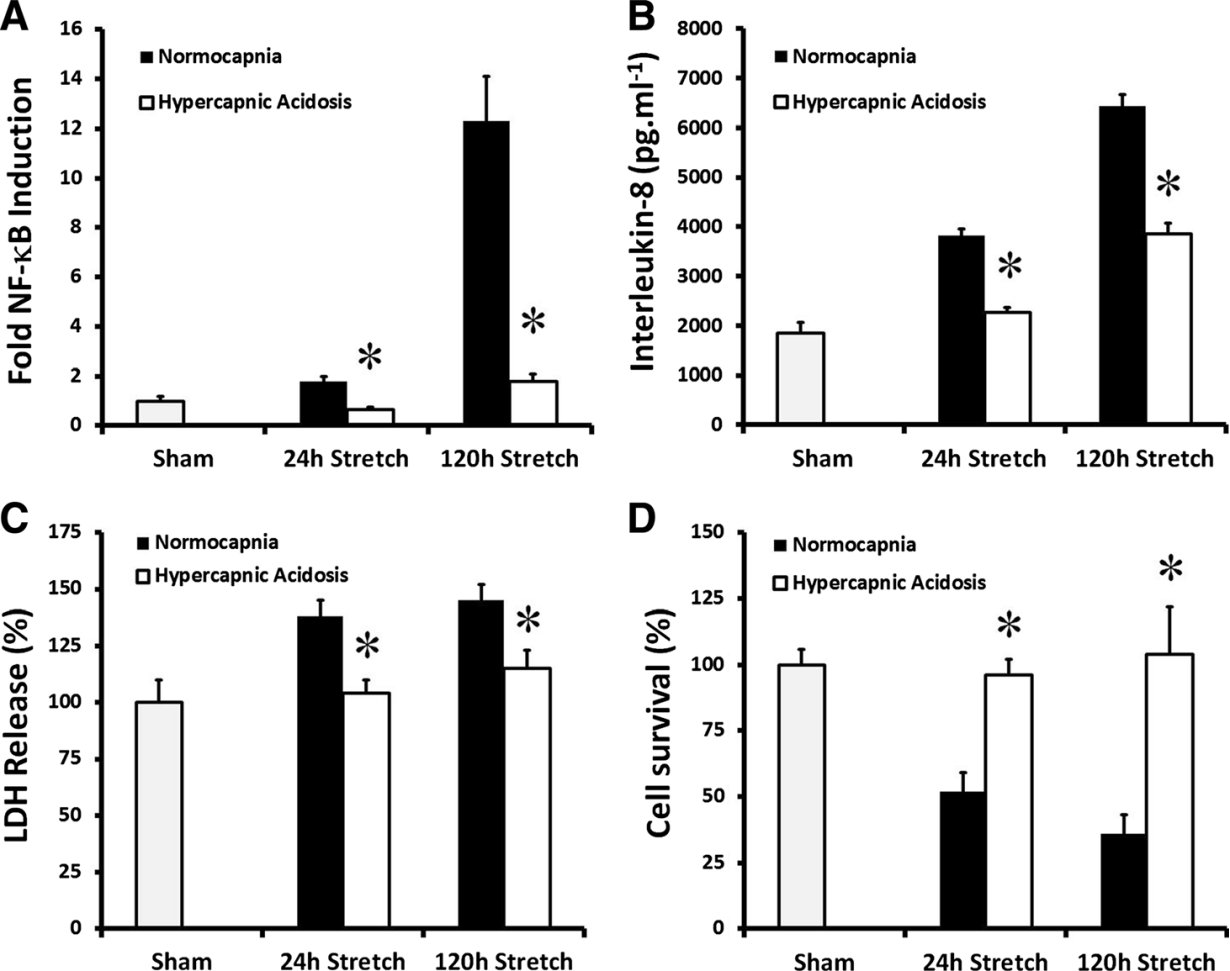
HCA attenuates alveolar epithelial injury following 24 and 120 h of cyclic mechanical stretch. HCA decreased stretch-induced activation of NF-κB (**a**), attenuated IL-8 secretion (**b**), reduced epithelial LDH leakage (**c**), and maintained cell viability (**d**) following 24 and 120 h of cyclic mechanical stretch in A549 alveolar epithelial cells. *Note:* *P < 0.05 versus normocapnia for each condition. Abbreviations: *Sham* unstretched cells

Cyclic stretch-induced a progressive activation of NF-κB at 24 and 120 h in A549 epithelial layers. HCA potently inhibited stretch-induced NF-κB activation at each time point (Fig. 2a). Cyclic stretch enhanced alveolar epithelial IL-8 secretion at 24 and 120 h, and this was attenuated by HCA at each time point (Fig. 2b). HCA abolished cyclic stretch-induced cell membrane injury at each time point (Fig. 2c). The progressive decrease in cell survival induced by cell stretch at 24 and 120 h was also abolished by HCA (Fig. 2d).

### Effect of pre- versus post-conditioning with HCA

**Fig. 3:**
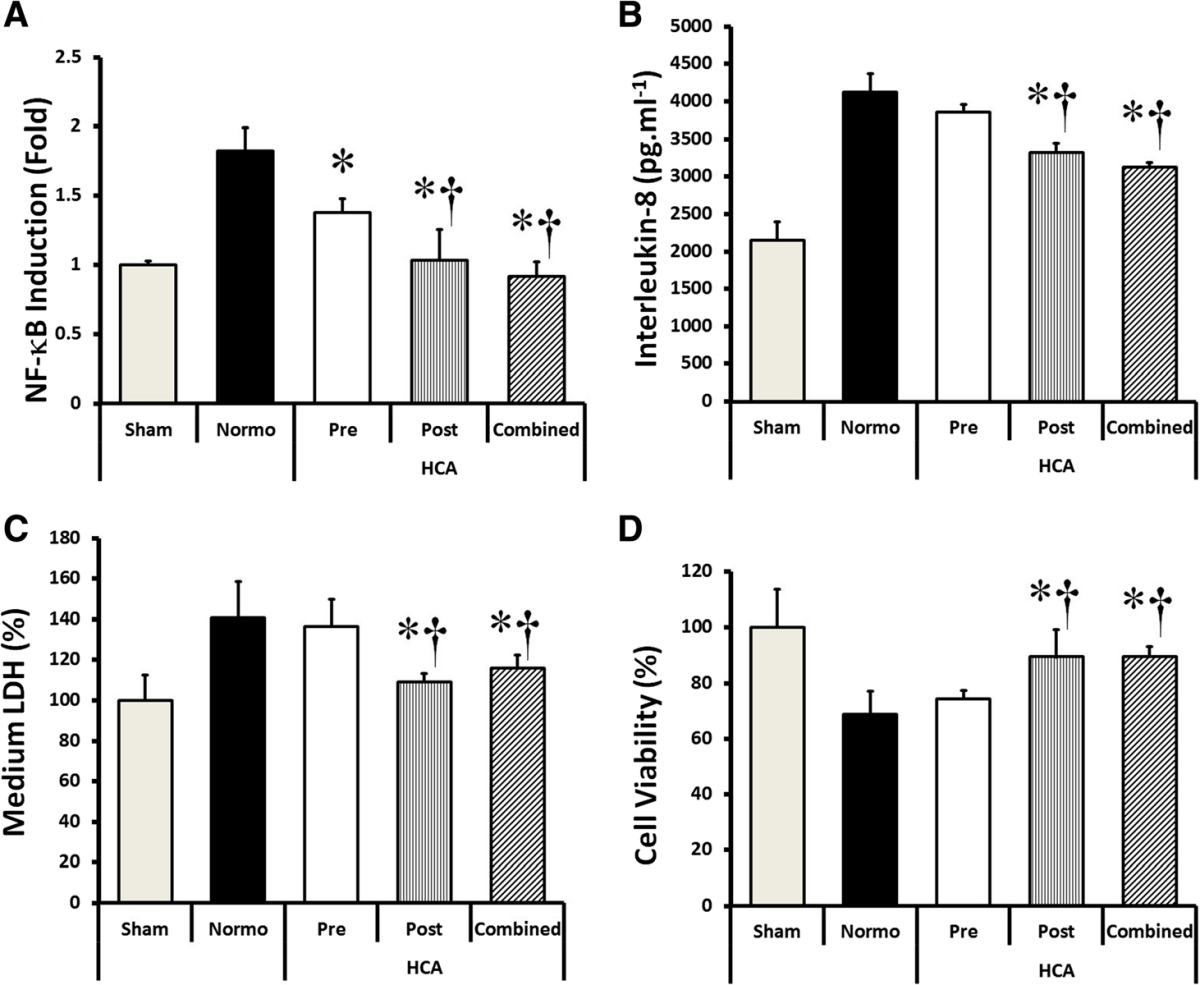
Effect of pre- versus post-conditioning with HCA. Pre-conditioning with HCA modestly decreased NF-κB activation (**a**) but did not alter the effect of high stretch on IL-8 production (**b**), LDH leakage (**c**), or cell survival (**d**). In contrast, exposure of the epithelial layers to HCA post-commencement of cell stretch was as effective as exposure to HCA prior to and post-commencement of cyclic stretch. *Note:* *P < 0.05 versus normocapnia; ^†^ *P* < 0.05 versus HCA-pre-conditioning. Abbreviations: *Sham* unstretched cells under normocapnia, *Normo* high cyclical stretch with normocapnia, *HCA-pre* hypercapnia exposure prior to cell stretch, *HCA-post* hypercapnia exposure commenced after start of cell stretch, *HCA-combined* hypercapnia exposure both prior to and during cell stretch high cyclical stretch with hypercapnia

Pre-conditioning with HCA was less effective compared to post-conditioning. Pre-conditioning modestly decreased NF-κB activation (Fig. 3a), but did not alter the effect of high stretch on IL-8 production (Fig. 3b), LDH leakage (Fig. 3c), or cell survival (Fig. 3d). In contrast, exposure of the epithelial layers to HCA post-commencement of cell stretch had the same effect as exposure to HCA prior to and post-cyclic stretch.

### HCA inhibits stretch-induced canonical NF-κB activation

#### HCA attenuates stretch-induced IκBα decrease

**Fig. 4:**
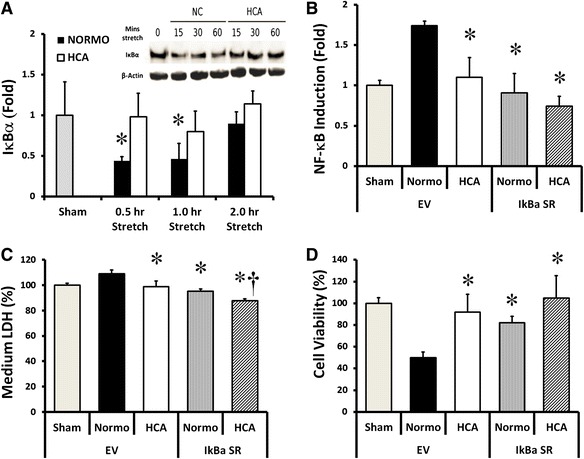
Effects of HCA on NF-κB pathway mediated via IκBα. ELISA with embedded Western blot demonstrating that HCA prevents the stretch-induced decrement in cytoplasmic concentrations of the NF-κB inhibitor IκBα (**a**). Direct overexpression of IκBα attenuates the deleterious effects of stretch in normocapnia-exposed epithelial layers, decreasing NF-κB activation (**b**), reducing epithelial LDH leakage (**c**) and preserving cell viability (**d**). HCA did further reduce medium LDH but had no effect on NF-κB or on cell viability, in the presence of prior IκBα overexpression. *Note:* *P < 0.05 versus normocapnia-empty vector, ^†^ *P* < 0.05 versus normocapnia-IκBα-SR. Abbreviations: *Normocapnia-Sham* unstretched cells with normocapnia, *Normocapnia-Stretch* high cyclical stretch with normocapnia, *HCA-Sham* unstretched cells with normocapnia, *HCA-Stretch* high cyclical stretch with hypercapnia

Cyclic epithelial stretch decreased cytosolic concentrations of the IκBα protein, which normally binds and inactivates NF-κB, at 15, 30, and 60 min, with restoration at 120 min (Fig. 4a). This decrease in cytosolic IκBα was abolished by HCA (Fig. 4a).

#### IκBα “occludes” the effect of HCA

Overexpression of IκBα directly attenuated the increase in stretch-induced NF-κB activation (Fig. 4b). IκBα “occluded” the effect of HCA on NF-κB activation, in that there was no further decrease in NF-κB activation in the presence of HCA in cells overexpressing IκBα (Fig. 4b). IκBα overexpression directly attenuated the increase in stretch-induced cell injury, as measured by LDH concentrations, and largely, but not completely, “occluded” this protective effect of HCA (Fig. 4c). IκBα overexpression also directly attenuated the decrease in stretch-induced cell viability and again “occluded” this protective effect of HCA (Fig. 4d).

### Acidosis versus CO_2_ in stretch-induced epithelial injury

**Fig. 5:**
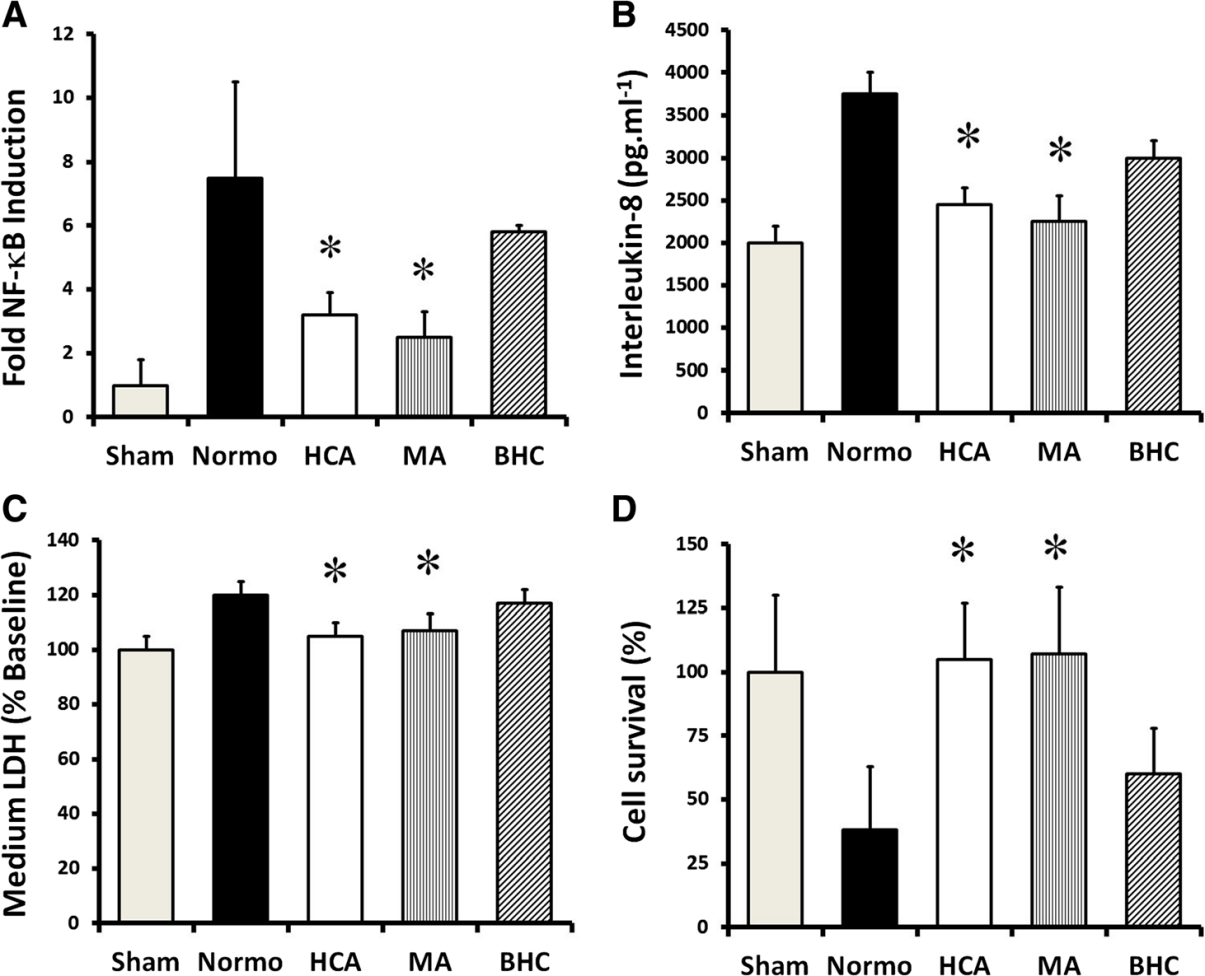
The effects of HCA appear to be mediated via pH-dependent effects rather than via CO_2_. Metabolic acidosis (MA), at a pH comparable to that seen with HCA, decreases stretch-induced activation of NF-κB (**a**), decreases IL-8 secretion (**b**), reduces epithelial LDH leakage (**c**), and maintains cell viability (**d**) following cyclic mechanical stretch. In contrast, these effects are abrogated by buffering of the hypercapnic acidosis (BHC). *Note:* *P < 0.05 versus normocapnia. Abbreviations: *Sham* unstretched cells under normocapnia, *Normo* high cyclical stretch with normocapnia, *HCA* high cyclical stretch with hypercapnia, *MA* high cyclical stretch with metabolic acidosis, *BHC* high cyclical stretch with buffered hypercapnia

Cyclic stretch activated NF-κB at 72 hours in A549 epithelial layers (Fig. 5a). Both HCA and metabolic acidosis (MA), but not buffered hypercapnia (BHC), inhibited this stretch-induced NF-κB activation (Fig. 5a). Cyclic stretch-induced alveolar epithelial IL-8 secretion was attenuated by acidosis—both hypercapnic and metabolic—but not by BHC (Fig. 5b). Both HCA and MA abolished cyclic stretch-induced cell membrane injury but BHC was not protective (Fig. 5c). The decrease in cell survival induced by cell stretch was also abolished by acidosis—both HCA and MA—but BHC was ineffective (Fig. 5d).

## Discussion

HCA is a consequence of respiratory failure and is particularly frequent in critically ill patients managed with conventional “protective” ventilation strategies. NF-κB is an important transcription factor that is activated in the setting of inflammation and repair and is central to innate immunity [31]. Ventilatory strategies incorporating permissive HCA have been shown to enhance patient survival in a clinical setting of ARDS by alleviating stretch-induced injury [3, 32–34]. While the associated HCA is generally considered a bystander, work from our group and others has demonstrated the potential for HCA to exert potent biologic effects in preclinical lung injury models, with several key effects mediated via inhibition of NF-κB signaling. This current work demonstrates that HCA reduces stretch-induced bronchial and alveolar epithelial cell NF-κB activation and consequent inflammation, injury, and cell death. These protective effects of HCA are mediated via the inactivation of the canonical NF-κB pathway and appear to be mediated by the acidosis rather than the CO_2_ per se.

### Deleterious effects of excessive cell stretch

Our findings confirm the deleterious effects of prolonged excessive cell stretch on the alveolar epithelium. Positive pressure ventilation can severely injure the lungs of already compromised ARDS patients, and here, we show that excessive cell stretch severely injures both bronchial and alveolar cells by activation of NF-κB, a finding which confirms and expands on previously published data [27]. Interestingly, our work also shows that HCA can alleviate this cell injury and inflammation following both short- and long-term stretch injury, an important finding from a clinical perspective, suggesting that permissive/therapeutic hypercapnia could be of benefit to the patient throughout the ventilation period.

### Protective effects of HCA in cell stretch injury

Furthermore, our findings demonstrate that HCA directly attenuates cell stretch-induced injury. HCA reduced stretch-induced bronchial and alveolar epithelial stretch-induced inflammation, as evidenced by pulmonary IL-8 secretion. HCA also attenuated cellular injury and maintained cell membrane integrity, as evidenced by reduced concentrations of LDH, an intracellular enzyme which is released into the medium during cell disruption or necrosis. HCA also maintained cellular viability, preventing stretch-induced cell death.

### HCA attenuates stretch-induced NF-κB activation

In this study, we have shown that mechanical stretch triggers the NF-κB signaling pathway. NF-κB activation in turn results in epithelial inflammation, loss of cell membrane integrity, and ultimately in cell death. When IκBα is directly overexpressed in the epithelial layers, it reduced stretch-induced NF-κB activation, decreased epithelial IL-8 production, maintained cell membrane integrity, and reduced stretch-induced cell death, demonstrating that these deleterious effects of cyclic stretch are mediated via NF-κB activation. HCA inhibited NF-κB activation, decreasing the detrimental effects of stretch injury.

### Mechanism by which HCA inhibits NF-κB pathway

IκBα is normally located in the cytoplasm, where it binds to and inactivates NF-κB. When the canonical NF-κB pathway is activated, the IKK-complex phosphorylates IκBα, which is then subsequently degraded. In these studies, HCA blocked the stretch-induced decrease of cytosolic concentrations of IκBα. In subsequent studies, the effect of HCA was comparable to that seen with direct IκBα overexpression, while the combination of IκBα overexpression and HCA was also comparably effective. Overexpression of IκBα greatly reduced or entirely abrogated the effect of HCA on stretch-induced inflammation, cell membrane dysfunction, or cell viability. This suggests that IκBα “occluded” the effect of HCA, suggesting that these effects of HCA are mediated largely via inhibition of the canonical NF-κB pathway.

These data support and extend prior findings by our group and others. Takeshita et al. showed HCA could inhibit endotoxin-induced canonical NF-κB activation and IκBα degradation in human pulmonary artery endothelial cells [30]. We have recently demonstrated that HCA directly reduces the kinase activity of the IKK complex and reduces IKK-mediated phospho-inactivation of IκBα [28], a finding entirely consistent with the findings from these studies. Taken together, these findings suggest that inhibition of the IKK-IκBα interaction constitutes a key mechanism by which HCA suppresses canonical NF-κB activation in response to stimuli such as endotoxin or high stretch.

### Role of acidosis versus CO_2_

The beneficial effects of HCA on stretch-induced pulmonary injury appear to be mediated by the pH change, i.e. the acidosis that is caused by the HCA rather than the increase in CO_2_ per se. In fact, buffering the HCA completely abolished its effect on the NF-κB activation pathway and abrogated the protective effects on inflammation, cell membrane leak, and cell viability. This contrasts somewhat with the findings of Takeshita et al., who demonstrated that buffered hypercapnia inhibited endotoxin-induced canonical NF-κB activation by suppressing IκBα degradation [30]. Furthermore, the inhibitory effects of HCA on wound healing, which were mediated at least in part via inhibition of NF-κB, appeared to be a function of the hypercapnia rather than the acidosis per se. Cummins et al., showed that hypercapnia inhibited endotoxin induced NF-κB activation in fibroblasts via activation of the non-canonical pathway [35]. Likely explanations for the divergent findings include the mechanism of injury—stretch versus endotoxin induced—and the cell types studied—epithelium versus endothelium and fibroblasts.

### Limitations

There are some of limitations to these studies. Our use of non-stretched cells as controls for these experiments was based on our demonstration that physiologic stretch did not produce any evidence of cell inflammation or injury (Additional file 2: Figure e1). While we have previously examined the effects of greater degrees of HCA than those used in the current study [27], we have not examined the efficacy of less severe HCA.

## Conclusions

We report that HCA enhances cell viability and protects against epithelial damage after early or prolonged stretch-induced injury by inhibiting canonical NF-κB activation through a pH-dependent mechanism. These findings further our knowledge in regard the effects of HCA on the NF-κB pathway and its effects in a mechanical stretch injury setting. The demonstration that direct inhibition of the NF-κB pathway is protective in other diverse pre-clinical non-septic ARDS models [24–26] but is potentially deleterious in sepsis-induced ARDS [36] underlines the importance of understanding the interaction between HCA and this pivotal pathway.

### Key message

Hypercapnic acidosis attenuates cyclic mechanical stretch-induced pulmonary epithelial inflammation, injury, and death via inhibition of stretch activation of canonical NF-κB pathway activation. This effect was pH rather than CO_2_ dependent. The precise mechanism by which HCA inhibits NF-κB activation appears to be via attenuation of stretch-induced phospho-inactivation of the cytosolic NF-κB inhibitor IκBα, thereby preventing the release of the activated NF-κB dimer.

## Additional files

Additional file 1: Figure S1. The IkBa-SR gene suppressed IL-1b and TNF-a activation of NF-kB. NF-κB activation induced by IL-1b and TNF-a activation (both 20 ng/ml) was attenuated by IkBa-SR gene overexpression in alveolar epithelial A549 cells. (JPG 72 kb) Additional file 2: Figure S2. Physiologic stretch does not cause pulmonary epithelial inflammation, injury or cell death. The application of 10 % equibiaxial cyclic stretch for 24 hours did not activate NF-kB (Panel A), increase interleukin-8 secretion (Panel B), alter membrane integrity as assessed by epithelial LDH leakage (Panel C), or affect cell viability (Panel D) in alveolar epithelial A549 cells, when compared to unstretched cells. (JPG 200 kb)
