# Supplementary figures and images for: Hypercapnic acidosis attenuates pulmonary epithelial stretch-induced injury via inhibition of the canonical NF-κB pathway
Source: Intensive Care Med Exp. 2016 Mar 22;4:8. doi: 10.1186/s40635-016-0081-6 (PMC4801837; doi:10.1186/s40635-016-0081-6)

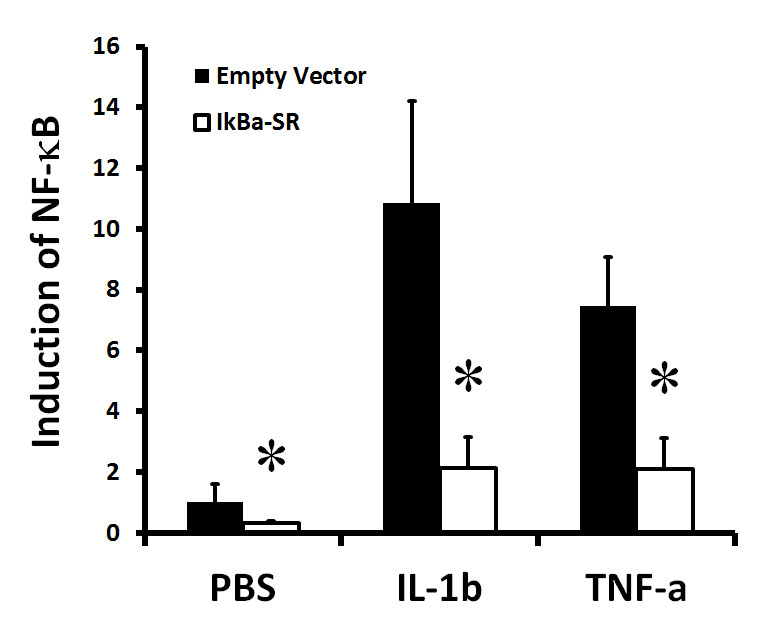

Supplement: Additional file 1: Figure S1. — The IkBa-SR gene suppressed IL-1b and TNF-a activation of NF-kB. NF-κB activation induced by IL-1b and TNF-a activation (both 20 ng/ml) was attenuated by IkBa-SR gene overexpression in alveolar epithelial A549 cells. (JPG 72 kb) [file 40635_2016_81_MOESM1_ESM.jpg]

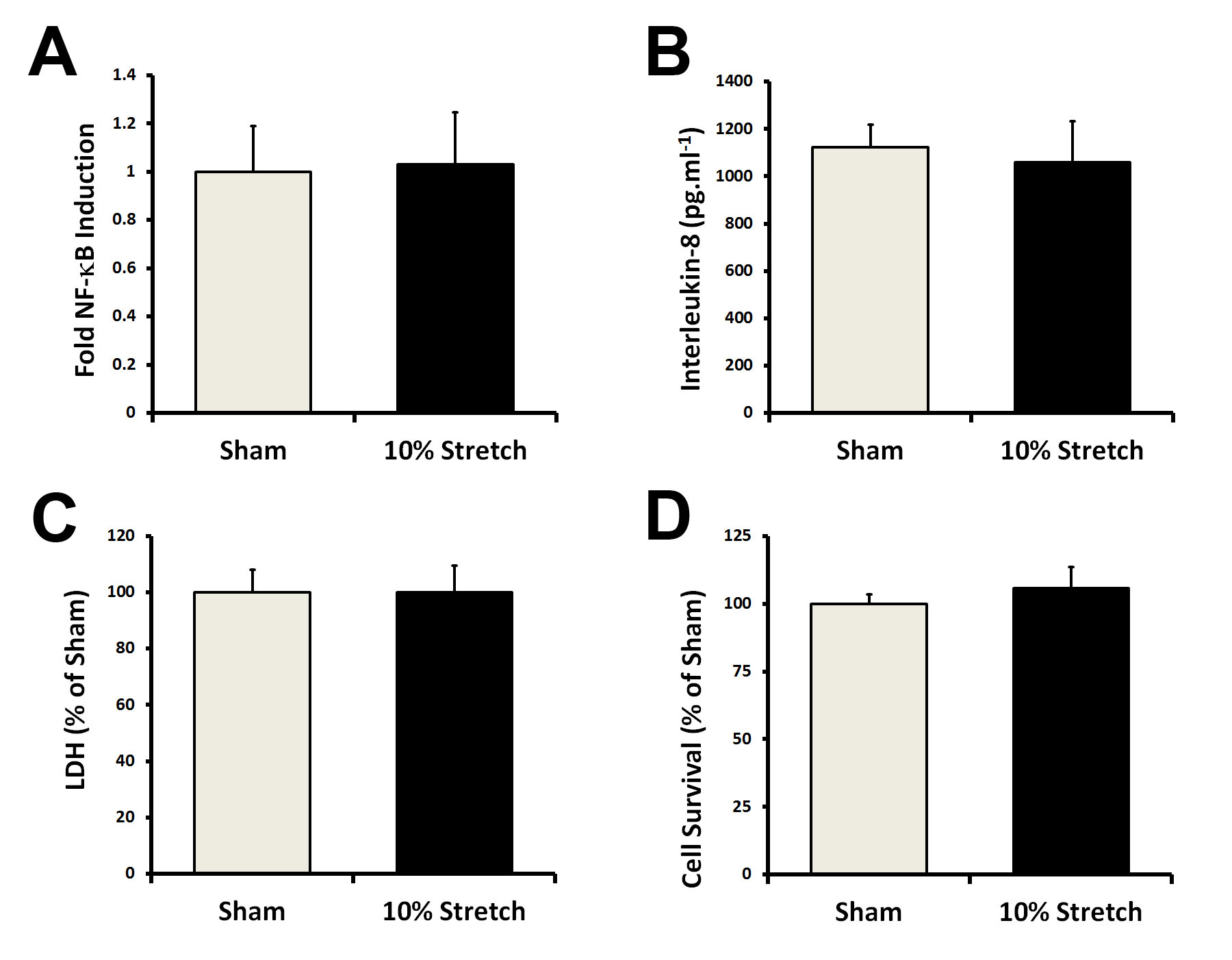

Supplement: Additional file 2: Figure S2. — Physiologic stretch does not cause pulmonary epithelial inflammation, injury or cell death. The application of 10 % equibiaxial cyclic stretch for 24 hours did not activate NF-kB (Panel A), increase interleukin-8 secretion (Panel B), alter membrane integrity as assessed by epithelial LDH leakage (Panel C), or affect cell viability (Panel D) in alveolar epithelial A549 cells, when compared to unstretched cells. (JPG 200 kb) [file 40635_2016_81_MOESM2_ESM.jpg]
